# Young Men’s Shame about Their Desire for other Men Predicts Risky Sex and Moderates the Knowledge – Self-Efficacy Link

**DOI:** 10.3389/fpubh.2014.00183

**Published:** 2014-10-20

**Authors:** Mina Park, Janeane N. Anderson, John L. Christensen, Lynn Carol Miller, Paul Robert Appleby, Stephen John Read

**Affiliations:** ^1^Annenberg School for Communication and Journalism, University of Southern California, Los Angeles, CA, USA; ^2^Department of Communication, University of Connecticut, Storrs, CT, USA; ^3^Department of Psychology, University of Southern California, Los Angeles, CA, USA

**Keywords:** young MSM, young adult, shame, self-efficacy, safe sex, sex behaviors, safe-sex knowledge, HIV/AIDS

## Abstract

**Background:** Nationally, HIV incidence is rising rapidly among young (18–24 years old) men who have sex with men (YMSM). Knowledge of safer sex generally enhances self-efficacy for safer sex, an important predictor of safer-sex behaviors. Recent findings suggest that a strong negative *social* emotion (i.e., shame) increases YMSM’s sexual risk-taking. Unchangeable shame (e.g., desire for other men) might undermine (moderate) the link between knowledge and self-efficacy or between self-efficacy and unprotected anal intercourse (UAI): this may be less likely for changeable shame (e.g., shame about risky sexual behavior).

**Aim:** To test the hypotheses that shame (i.e., sexual desire shame), but not shame about behavior (i.e., sexual behavior shame), will be positively related to UAI and will moderate the relationship between knowledge and self-efficacy and/or self-efficacy and UAI among YMSM.

**Method:** In an online national study, 1177 young adult (18–24 years old) MSM reported one or more acts of UAI in the past 90 days with a casual partner. Eligible MSM filled out a survey in which they provided information about their knowledge of safer sex, self-efficacy for safer sex, reported levels of shame, and reported past 90-day UAI.

**Results:** Sexual desire shame was negatively correlated with knowledge and self-efficacy and positively correlated with UAI, the pattern reversed for sexual behavior shame. Sexual desire shame significantly lowered the knowledge to self-efficacy and the self-efficacy to UAI links. Sexual behavior shame also reduced the link from knowledge to self-efficacy, but not the self-efficacy to UAI link.

**Conclusion:** The present study shows that there are different types of shame that may produce different effects with different implications for health behavior. Sexual desire shame may better reflect an emotion that is activated prior to risky behavior (e.g., when men reflect upon or feel desire for another man). Sexual behavior shame, on the other hand, better reflects what has already happened, thus, those higher in knowledge, efficacy, and therefore, safer sex are least likely to experience shame behavior.

## Introduction

Men who have sex with men (MSM) not only have the highest HIV incidence (i.e., number of new infections) of any subpopulation in the United States (1) but also they are experiencing the sharpest incidence *increase*. In 2010, MSM accounted for 63% of estimated new infections; in 2011, this group accounted for 69% of all HIV diagnoses (1). Furthermore, the highest incidence is reported for the youngest MSM, those 24 years of age or younger (1). HIV transmission is most likely to occur during unprotected anal intercourse (UAI), making this behavior “high risk” for STI/STD and HIV/AIDS transmission (2). Thus, prior work on sexual risk-taking among MSM has often focused on identifying factors that predict young MSM’s risky sexual decisions.

Theory-based HIV prevention interventions have proven successful at increasing HIV protective behaviors (e.g., increased condom use, reduction in the number of sex partners) among the general population (3). Interventions that are most effective at achieving desired outcomes are ones that contain attitudinal components, education information, and behavioral skills training (4). HIV-risk-reduction interventions targeted to MSM populations have demonstrated effectiveness by increasing participants’ knowledge of safer sex and perceptions of self-efficacy (5, 6). Past research has indicated a relationship between self-efficacy and safer-sex behavioral practices (5). Research suggests MSM who believe they are better able to protect themselves (i.e., have greater self-efficacy) report lower rates of unsafe sex than those with lower self-efficacy (6, 7). Furthermore, prior work suggests knowledge generally enhances self-efficacy (8, 9), and HIV-related knowledge increases safer-sex self-efficacy, as well as HIV-risk reduction (10–17). Based on this, many HIV-risk-reduction interventions for MSM have been designed to increase both safer-sex knowledge and self-efficacy (18, 19).

Many MSM consistently engage in safer sex. However, despite known risks and interventions, a significant proportion of young MSM still report risky sex – including insertive and receptive UAI (3, 4). Greater understanding of factors that predict MSM’s risky sexual decisions is critical, especially for subgroups of younger men who have sex with men (YMSM) (i.e., those between 18 and 24 years of age) currently engaging in UAI.

Recently, researchers have suggested that a variety of social factors (e.g., stigma, shame) may be undermining HIV prevention efforts and fueling the AIDS epidemic among MSM (20–23). Shame is a negative affective reaction focused on one’s entire self as blameworthy, flawed, and unchangeable. The catalyst for shame-induced self-scrutiny is an objectionable behavior that is perceived as reflecting on the objectionable self (22). “In shame, the self is both agent and object of observation and disapproval, as shortcomings of the defective self are exposed before an internalized observing ‘other”’ (p. 1257). MSM may internalize negative feelings or attitudes related to same-sex sexuality, which adversely impacts safer-sex behaviors “in the moment.” In line with this, Christensen and colleagues (24) found that shame [as measured by a subscale of the PANAS-X (23)] predicts UAI in high-risk young MSM populations.

Although reliable and widely used, the PANAS-X subscales are broad, non-specific measures of affect. As such, the measure does not provide insight regarding the determinants of shame, which are likely to vary across MSM. On the one hand, shame may be caused by the realization that one has violated a personal standard by engaging in *risky sexual behavior*. A self-regulatory failure such as this can be changed (by using a condom during future sexual encounters). In addition to this sexual behavior shame, it is possible that young MSM feel sexual desire shame. This type of shame is related to stigma and arises from beliefs that their unchangeable *sexual desires* for other men are non-normative and unaccepted by society. For example, parents may not be as supportive of children with a homosexual versus heterosexual identity, and shame may inhibit young MSM’s self-acceptance (25). Furthermore, peers frequently victimize sexual minority youth because of their identity (26).

Christensen and colleagues (24) argued that perhaps sexual desire shame and that inability to change desires (e.g., versus ability to change risky behavior) is what creates self-regulatory problems that lead to risk-taking. Thus, MSM may not adequately address those feelings (e.g., desires for other men) that may be in conflict with other beliefs (e.g., desiring other men is bad). On the other hand, neuroscience research suggests that negative affect about changeable behavior (i.e., risk-taking) might actually reduce the probability of engaging in that behavior again (27). Thus, shame about changeable behavior would not be similarly expected to increase risk-taking. But, Christensen et al. (24) did not examine whether sexual desire shame, or sexual behavior shame, was related to PANAS-X shame (found to positively predict UAI). We examine these links in the current work.

The relationships among knowledge, self-efficacy, and UAI have been established in extant literature. However, we lack a more nuanced understanding of the impact of shame on risky sexual behaviors (i.e., UAI), particularly whether young MSM’s shame about their sexual desires for same-sex partners moderates the knowledge to self-efficacy or the self-efficacy to UAI link, making it difficult for interventions that increase knowledge or self-efficacy to reduce UAI. For example, when individuals experience shame they may have difficulty integrating new knowledge into their sex-related behavioral routines. This may serve to decrease their feelings of confidence in negotiating condoms during sex, undermining attempts at safer sex.

Based on the previous literature, we would predict the following. First, in line with the logic of Christensen et al. (24), we would predict that PANAS-X shame is positively related to sexual desire shame but not sexual behavior shame. Second, sexual desire shame (but not sexual behavior shame) is expected to negatively correlate with knowledge and self-efficacy and positively predict UAI. Third, sexual desire shame (but not sexual behavior shame) will moderate the knowledge to self-efficacy link, attenuating (lowering) the strength of that link. Fourth, sexual desire shame (but not sexual behavior shame) will also moderate (lower) the self-efficacy to UAI link.

## Materials and Methods

### Designs and setting

The cross-sectional data presented are from survey baseline data from the socially optimized learning in virtual environments (SOLVE) project that used a nationwide online sample of high-risk, young MSM from the United States. SOLVE interventions have been developed to reduce sexual risk-taking among MSM using virtual guides/virtual self characters (28–31). This overall project was designed to develop and test the effectiveness of SOLVE nationally over the web and was funded by the National Institute of Mental Health (NIMH). From February 12, 2012 to October 28, 2012, members of the target population were reached by clickable banner ads on Craigslist, gay interest websites, and a project-related Tumblr blog. Potential respondents accessed the project website to fill out an eligibility screener.

### Ethical considerations

This study was approved by the Internal Review Board (IRB) of the University of Southern California. Informed consent was a necessary pre-condition for participant enrollment. Participants were included in the study only after informed consent was obtained. Participants were assured that their responses would be confidential and told that their participation was voluntary, meaning they could withdraw from the study at any point or refuse to answer questions that they were not comfortable answering.

### Participants

Eligible men were those who (1) reported they had received an HIV-negative test result, (2) lived in the United States, (3) were between 18 and 24 years of age, and (4) engaged in UAI in the 3 months prior to the study with a non-primary male partner (i.e., a man with whom the participant was not currently in a romantic relationship). After eligibility was determined, participants read the consent form and decided whether to participate in the study or not. Participants had a 1:40 chance of receiving a $100 gift card when they enrolled in this study. Here, the data come from 1177 participants who enrolled in the project between February and November 2012 and filled out baseline measures. The average age of participants was 21.26 (SD = 1.84). Most had earned a post-secondary degree or had at least some college experience (77.9%), while 21.0% of participants reported an educational attainment equivalent to a high-school diploma or lower with the remaining participants refusing to respond. Regarding ethnicity, 72.5% of participants identified themselves as White/Caucasian, while 14.9 and 12.6% of participants self-identified as Hispanic/Latino and Black/African-American, respectively. Participants were asked their sexual orientation and most of participants identified themselves as Gay/Homosexual (76.3%), followed by Bisexual (14.8%). Only the baseline measures are analyzed here, although the overall study involved a longitudinal randomized controlled trial aimed at reducing shame, the results of which are reported elsewhere (24).

### Measures

#### Self-efficacy for safer sex

In order to measure MSM’s self-efficacy for safer sex, three items from the self-efficacy for HIV-preventive behavior scale developed by Kalichman and Nachimson (32) were used. On the basis of social cognitive theory (33) and research on the assessment of self-efficacy for practicing safer sex (34, 35), measurement includes participants’ confidence in their ability to discuss safer sex in a risky-sex situation, ability to suggest using condoms with a sexual partner, and refusal of unsafe sex when pressured by the partner. Items administered in this study included “How confident are you that you could bring up the issue of condoms to your sexual partner?,” “How confident are you that you can convince your sexual partner that the two of you should use a condom or have safe sex, even if he says he does not want to?,” and “How confident are you that you would leave the situation if your sexual partner refused to use a condom?.” The scale ranged from 0 (can not do at all) to 10 (certain can do), and the present study used the average of those items as indicators of self-efficacy for safer sex (Cronbach’s α = 0.78, *M* = 7.55, SD = 1.96).

#### Knowledge of safer sex

Participants were asked to indicate to what extent they agreed with 25 statements regarding safe-sex behaviors with male sexual partners. The items ranged from more general (e.g., “It is a good idea to talk about safer sex before you go into the bedroom”) to more specific statements (“Having oral sex is lower risk for HIV than anal sex”). The responses were recorded on a 5-point scale that ranged from 1 (strongly disagree) to 5 (strongly agree) and were summed into a composite (Cronbach’s α = 0.81, *M* = 3.47, SD = 0.57). High scores indicated greater knowledge. The knowledge items were devised for this project based on the specific knowledge items that the intervention was designed to change, and they specifically mapped on to what one needed to know to successfully negotiate safer sex. This measure of knowledge (of what to do, why) parallels the measure of self-efficacy for safer sex above (i.e., confidence in successfully negotiating safer sex).

#### Shame

To examine whether the extent to which PANAS-X shame is correlated with sexual desire and sexual behavior shame, we included a measure of PANAS-X shame. Participants were asked to indicate to what extent they have felt shame during the past few weeks. The scale values range from 1 (very slightly or not at all) to 5 (extremely). This measure of shame has been extensively used in the past (23), has high reliability (Cronbach’s α = 0.88), and it was previously reported that PANAS-X shame predicts UAI for MSM in this sample (24).

To examine whether shame moderates the effects of knowledge of safer sex on self-efficacy for safer sex and the effect of self-efficacy on UAI, we measured shame about sexual desire for other men using two items that were not part of the PANAS-X shame measure: “When I think about my desire for other men, I feel like a bad person” and “I feel bad about my desire for other men.” Participants used a scale ranging from 1 (strongly disagree) to 7 (strongly agree) to indicate the degree to which they agreed with the shame-related statements. According to this scaling, high scores would indicate high-shame levels. The measure has high reliability (Cronbach’s α = 0.94). The average score of the two shame items used in the final analysis was *M* = 3.67 (SD = 1.80). In addition to sexual desire shame, we measured shame about prior sexual risk behavior using two items: “When I think about the times I have had unsafe sex with other men, I feel like a bad person” and “I feel bad about having unsafe sex with other men.” The values on this measure similarly ranged from 1 (strongly disagree) to 7 (strongly agree) and the measure has high reliability (Cronbach’s α = 0.88). The average score of the two sexual risk shame items was *M* = 4.38 (SD = 1.27). The two measures of shame have a modest correlation (*r* = 0.084, *p* = 0.006).

#### Unprotected anal intercourse

To examine whether shame moderates the effects of self-efficacy for safer sex on UAI, we measured UAI using two items: “How many times in the past 90 days did you engage in anal sex as a TOP with all male non-primary partners and primary partners?” and “How many times in the past 90 days did you engage in anal sex as a BOTTOM with all non-primary partners and primary partners?.” Participants were asked to type the number of acts of UAI. As is often the case with sexual count data, the variances of responses were large (UAI top: mean = 4.95, median = 3.00, SD = 5.90; UAI bottom: mean = 4.38, median = 2.00, SD = 7.64; UAI overall: mean = 8.90, median = 6.00, SD = 13.87) and these three variables were significantly skewed (*z* score: −0.840 ~ 11.026 (UAI top); −0.573 ~ 12.522 (UAI bottom); −0.642 ~ 11.614 (UAI overall). Because the patterns for UAI top and UAI bottom were similar across analyses, these were collapsed into a single measure of UAI overall.

### Statistical analysis

All data were analyzed using the Statistical Package for the Social Sciences (36). First, to test if PANAS-X shame is related to sexual desire shame, but not sexual behavior shame, we performed Spearman correlations, designated by *r_s_* because measures of PANAS-X shame are significantly skewed (37). The second hypothesis was that sexual desire shame (but not sexual behavior shame) would be significantly correlated with knowledge of safer sex (negatively), self-efficacy for safer sex (negatively), and UAI (positively): Pearson correlations, designated by *r*, were used for the not significantly skewed variables and Spearman correlations, designated by *r_s_* were used for correlations with UAI since UAI is a significantly skewed variable. The third hypothesis was that sexual desire shame (but not sexual behavior shame) may moderate the knowledge to self-efficacy link, attenuating that link. To test this, hierarchical regression was performed. The last hypothesis was that sexual desire shame (but not sexual behavior shame) moderates the self-efficacy to UAI link, lowering that link. To test this last moderation hypothesis, negative binomial regression analyses were employed because the dependent variable in this study (counts of UAI) is significantly skewed. A statistically significant interaction effect for these moderation hypotheses would provide evidence that the impact of knowledge of safer sex on self-efficacy for safer sex or the impact of self-efficacy for safer sex on UAI is dependent upon the level of shame reported by participants (38). Alpha was set to *p* = 0.05. Due to missing data, the sample size varies for each individual analysis.

## Results

First, in line with the logic developed in Christensen et al. (24), we predicted that sexual desire shame (but not sexual behavior shame) would be positively related to the PANAS-X general measure of shame. Consistent with that prediction, PANAS-X general shame is significantly related to sexual desire shame (*r_s_* = 0.312, *p* < 0.001), but not sexual behavior shame (*r_s_* = 0.025, *p* = 0.421) (see Table 1). Thus, the first hypothesis was supported.

**Table 1:** **Correlation matrix of shame, knowledge, self-efficacy, and unprotected anal intercourse (UAI)**.

	1	2	3	4	5	6
	Desire shame	Behavior shame	PANAS-X shame	Knowledge	Self-efficacy	UAI
1. Sexual desire shame	–					
2. Sexual behavior shame	0.116***	–				
3. PANAS-X shame	0.31***	0.03	–			
4. Knowledge	−0.26***	0.14***	−0.28***	–		
5. Self-efficacy	−0.55***	0.09**	−0.17***	0.49***	–	
6. UAI	0.41***	−0.21***	0.19***	−0.54***	−0.341***	–

*Correlations with PANAS-X shame and UAI are Spearman correlations, all others are Pearson correlations. *N* = 399 ~ 437 for UAI correlations; *N* = 969 ~ 1,160 for other correlations*.

***p < 0.001; **p < 0.01.

In order to determine whether sexual desire shame or sexual behavior shame is related to knowledge of safer sex, self-efficacy for safer sex, and UAI, we assessed the correlations among these variables. The results indicate that sexual desire shame was negatively correlated with knowledge of safer sex (*r* = −0.259, *p* < 0.001) and self-efficacy for safer sex (*r* = −0.554, *p* < 0.001), while sexual behavior shame was positively correlated with knowledge of safer sex (*r* = 0.135, *p* < 0.001) and self-efficacy for safer sex (*r* = 0.091, *p* = 0.003). Regarding UAI, sexual desire shame, like PANAS-X shame, positively predicted UAI (*r_s_* = 0.414, *p* < 0.001). On the other hand, sexual behavior shame showed exactly the opposite pattern, it was negatively related to UAI (*r_s_* = −0.208, *p* < 0.001) (see Table 1). Thus, the second hypothesis was supported.

Although sexual desire shame and knowledge of safer sex both are significantly correlated with self-efficacy for safer sex, when they are considered together in the same regression (see Model 1 in Table 2), sexual desire shame is no longer a significant predictor, suggesting that it shares considerable variance with knowledge of safer sex. Model 2 directly tests our third hypothesis: sexual desire shame (but not sexual behavior shame) will moderate the knowledge to self-efficacy link, attenuating the strength of that link. Consistent with it, we found sexual desire shame moderates the relationship between knowledge and self-efficacy for safer sex (coefficient = −0.17, SE = 0.06, *p* = 0.003). Table 2 shows that the interaction between shame and knowledge of safer sex accounts for a statistically significant portion of the variance (incremental *R*^2^ = 0.5%, *p* = 0.003). In other words, the magnitude of the relationship between knowledge and self-efficacy varies by level of shame.

**Table 2:** **Hierarchical regression predicting self-efficacy for safer sex**.

	Model 1		Model 2	
	B (SE)	β	B (SE)	β
Knowledge of safer sex	1.70 (0.11)***	0.50	1.71 (0.11)***	0.51
Sexual desire shame	0.03 (0.03)	0.03	0.06 (0.06)	0.06
Incremental *R*^2^ (%)	23.5***			
Interaction				
Sexual desire shame × knowledge of safer sex			−0.17 (0.06)*	−0.08
Incremental *R*^2^ (%)			0.5**	
Total *R*^2^ (%)			24.0***	

*All entries with interaction terms are centered at the mean (38). *N* = 1,036*.

*p < 0.05; ***p < 0.001.

**Figure 1:**
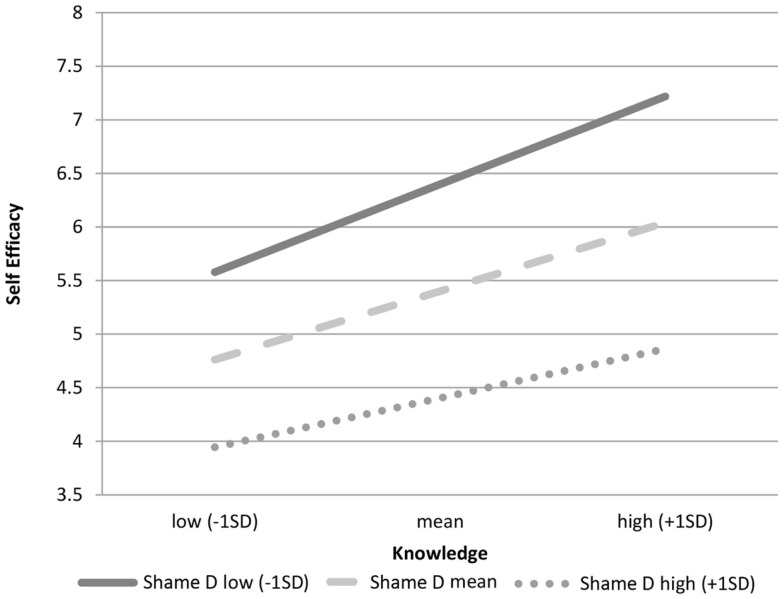
**Role of knowledge on self-efficacy by levels of sexual desire shame**.

We explored the nature of this interaction by estimating regression equations when shame was low (−1 SD), medium (mean), and high (+1 SD) following Aiken and West (39). Figure 1 shows that the slope of the line for low shame is much steeper than the slopes of the line for high shame. This result suggests that each increment in knowledge for low-shame MSM results in a greater increment in self-efficacy than it does for high-shame MSM.

**Figure 2:**
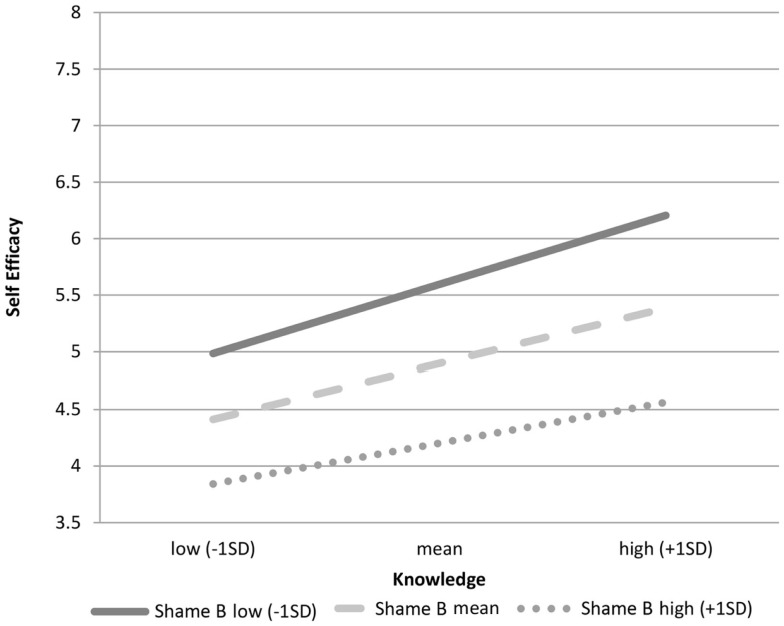
**Role of knowledge on self-efficacy by levels of sexual behavior shame**.

We compared the above result for sexual desire shame with the results using sexual behavior shame as the moderator. Unlike the prediction, results show that sexual behavior shame is also moderating the relationship between knowledge of safer sex and self-efficacy for safer sex (coefficient = −0.17, SE = 0.07, *p* = 0.019). Thus, the third hypothesis was partially supported. However, Figure 2 suggests that the effect for shame about behavior is weaker than that for shame about desire.

**Figure 3:**
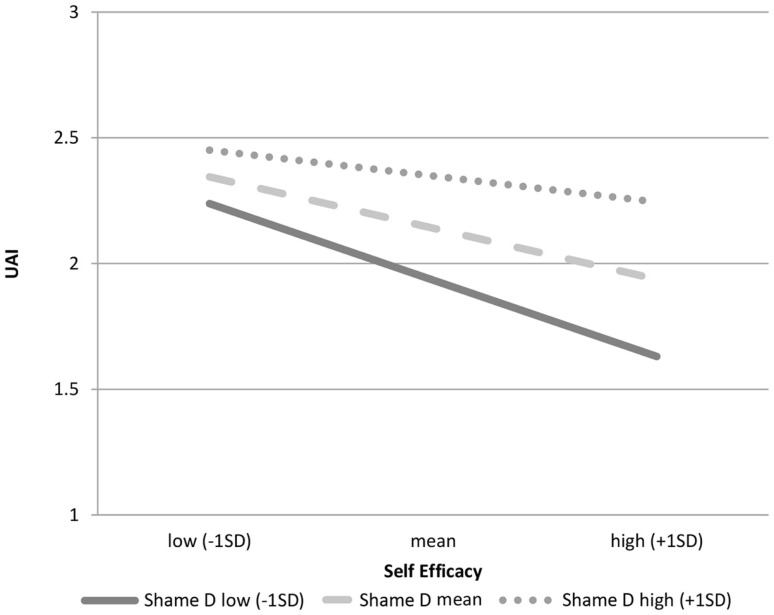
**Role of self-efficacy on UAI by levels of sexual desire shame**.

In the fourth hypothesis, we predicted that sexual desire shame may also moderate the self-efficacy to UAI link. Negative binomial analysis shows that sexual desire shame is a marginally significant moderator of the relationship between self-efficacy and UAI (coefficient = 0.027, SE = 0.01, *p* = 0.063, *N* = 432). Figure 3 shows that the slope of the line for low shame is steeper than the slopes of the line for high shame, suggesting that each increment in self-efficacy for low-shame MSM results in a greater decrease in UAI overall than it does for high-shame MSM. Sexual behavior shame, unlike sexual desire shame, was not a promising moderator to the link between self-efficacy for safer sex and UAI (coefficient = −0.032, SE = 0.02, *p* = 0.103, *N* = 397). Thus, the fourth hypothesis was supported.

## Discussion

This is the first work to show that there are different types of shame that may produce different effects with different implications for health behavior. Sexual desire shame involves men’s shame about their desires for other men, such desires are *not changeable*. Sexual behavior shame involves men’s shame that they have engaged in a risky behavior (unsafe sex), but this is a behavior they *can change* in the future (i.e., they can have safe sex).

Sexual desire shame and sexual behavior shame predict very different patterns of relationships with UAI, self-efficacy for safer sex, and knowledge of safer sex. Sexual desire shame is negatively related to knowledge of safer sex and self-efficacy for safer sex and correspondingly positively related to UAI: this type of shame may increase risky sexual decision-making. Sexual behavior shame, on the other hand, is positively related to knowledge of safer sex and self-efficacy for safer sex and correspondingly negatively related to UAI. What explains these different patterns of sexual desire versus sexual behavior shame? Perhaps, sexual behavior shame better reflects what has already happened, thus, those higher in knowledge, efficacy, and therefore, safer sex are least likely to experience shame behavior. Sexual desire shame, on the other hand, may better reflect an emotion that is activated prior to risky behavior (e.g., when men reflect upon or feel desire for another man), sexual desire shame has greater potential for attenuating or undermining *both* the links between knowledge and self-efficacy and self-efficacy and UAI, making its reduction a clear target for enhancing intervention change. Still, any felt shame – including shame about past sexual behavior – may interfere with learning and with enhancing links from learning to new skills. Perhaps this might explain not only sexual shame’s moderation effect on this link but also the weaker, similar moderation effects for behavior shame.

Christensen et al. (24) speculated that the type of shame that might increase UAI was sexual desire shame. That is because sexual desire shame is not changeable, producing an unresolvable conflict. In addition to what is suggested above that conflict might detract from men’s ability to focus on negotiating safer sex (i.e., affecting their interpersonal negotiation capacity) or that might lead high-shame MSM to try to resolve their conflict in unhealthy ways (e.g., alcohol, drugs) that would in turn increase risk. The current work makes clear that some shame (i.e., sexual desire shame) can increase risk while other forms of shame (e.g., sexual behavior shame) are less likely or unlikely to do so.

Consistent with prior work (9), the current findings demonstrate that knowledge is an important variable in predicting self-efficacy often thought to be an important precursor to safe-sex behaviors (e.g., condom use during anal intercourse). Nevertheless, the benefits of knowledge in increasing self-efficacy are reduced for high- compared to low-sexual desire shame. The significant moderating effect of sexual desire shame on the link between knowledge and self-efficacy suggests that combating sexual desire shame is key to infection reduction among MSM.

Results from this study have important implications for public health officials charged with reducing HIV infection rates for MSM. In a recent intervention, Christensen et al. (24), conducting analyses with the same sample as in the current work, found that *shame reduction* (due to intervention components in a sex positive interactive video game) predicted reduction in UAI over 3 months. Christensen et al. (24) used the PANAS-X general shame measure – associated with the sexual desire shame measure but not the sexual behavior shame measure used here. They argued that reducing conflicts about sexual desire shame might reduce UAI over time for young at-risk MSM because it may make it more difficult for young MSM to process and integrate knowledge of safer sex, a frequently proffered forerunner of self-efficacy. Certainly, Christensen et al.’s (24) logic is consistent with the moderating role found here of sexual desire shame.

Our data are consistent with the idea that shame somehow undermines or interferes with the positive behavioral effects of cognitive, knowledge-based processing. But, what are the possible mechanisms that might explain this overall process? To answer this question, we turn to the cognitive escape model (40), a theoretical perspective that has been widely applied to HIV-risk behaviors. McKirnan and his colleagues hypothesize that many MSM find themselves engaging in risky sexual behavior, “not because they lack information or intentions to be safe, but because coping with HIV risk over time becomes aversive, and motivates them to escape self-awareness of HIV” (p. 660). The model posits that negative affect, such as shame, leads to an aversive reaction that results in cognitive disengagement. In turn, this cognitive disengagement causes people to rely *less* on controlled, deliberate thinking and prior knowledge when making sexual decisions. Instead, people tend to rely *more* on non-conscious scripts that guide behavior in an automatic way. Unfortunately, in the context of sexual decision-making, these automatic scripts tend to largely consist of HIV-risk behaviors because, as McKirnan and colleagues (40) argue, “many men find condom use to be inconvenient or even unpleasant, and the erosion of safer sex norms in some segments of the gay community can make the negotiation of condom use difficult” (p. 662–663). The cognitive escape model certainly provides an interesting perspective when attempting to identify possible causal mechanisms that explain the moderating role of shame observed in our dataset. In future work, we plan to further explore the role of cognitive disengagement as it relates to shame, knowledge, efficacy, and HIV-risk behavior.

This work should be interpreted in light of its limitations. The analyses reported here were based on cross-sectional data. In future work, we plan to examine these effects over time to assess whether similar patterns emerge. We chose in this work to use measures of knowledge that were more specific to successful or unsuccessful sexual negotiations. We did this to better map the type of knowledge one needs in sexually risky situations pertaining to “what one can do and why” to the relevant self-efficacy (i.e., one’s confidence in actually acting on that knowledge in sexual interactions). Nevertheless, our findings may not as readily generalize to all the work linking knowledge and self-efficacy in HIV prevention and other areas of public health. In addition, we plan to explore the role of sexual orientation on these relationships. Although there were no apparent differences in patterns reported in our study across MSM sexual orientation categories in preliminary analysis, shame is closely related to sexual orientation issue. The bulk of the sample in this study were men who self-identified as gas/homosexual and we plan to examine the effects of other sexual orientation in a more detailed way.

Regardless of these limitations, however, the study raises new questions about the role of shame in moderating links among knowledge, self-efficacy, and risk-taking. In future work, we need to better measure self-efficacy so we can determine when and how those judgments are accurate, underestimates, or overestimates of individuals’ abilities to successfully negotiate safer sex in risky and challenging contexts – and whether that matters. We also need to better measure and understand how different kinds of shame produce positive and negative effects on behavior change. Games – that have already demonstrated their potential value in changing risky behaviors – may also provide a way to create contexts similar to those in real life in which to systematically manipulate and examine what, when, how, and for whom contextual cues may activate different types of shame and how that in turn affects self-efficacy and sexual risk-taking both within virtual environments and in subsequent real-world behavior over time.

## Conclusion

The current work is the first to show that sexual desire shame (1) is positively related to UAI and negatively related to safer-sex knowledge and self-efficacy, (2) is a significant moderator that reduces the links between knowledge and self-efficacy, (3) is a promising moderator that reduces the links between self-efficacy and UAI, and (4) is distinct from sexual behavior shame that is not related to higher levels of UAI. Better understanding of how different types of shame operate in sexual decision making for different target audiences is an important new frontier in changing young MSM’s risky sexual decision-making.

## Conflict of Interest Statement

The work reported here was supported by the National Institute of Mental Health (Grant number R01MH092671). The authors declare that the research conducted did not involve any financial or commercial relationships that could be perceived as a potential conflict of interest.
